# Major TCR Repertoire Perturbation by Immunodominant HLA-B^*^44:03-Restricted CMV-Specific T Cells

**DOI:** 10.3389/fimmu.2018.02539

**Published:** 2018-11-14

**Authors:** Meriem Attaf, Amna Malik, Mai C. Severinsen, Julia Roider, Paul Ogongo, Søren Buus, Thumbi Ndung'u, Alasdair Leslie, Henrik N. Kløverpris, Philippa C. Matthews, Andrew K. Sewell, Philip Goulder

**Affiliations:** ^1^Division of Infection and Immunity, Cardiff University School of Medicine, Cardiff, United Kingdom; ^2^Systems Immunity Research Institute, Cardiff University, Cardiff, United Kingdom; ^3^Department of Paediatrics, University of Oxford, Oxford, United Kingdom; ^4^Africa Health Research Institute, Durban, South Africa; ^5^Laboratory of Experimental Immunology, Faculty of Health Sciences, University of Copenhagen, Copenhagen, Denmark; ^6^Department of infectious diseases, Medizinische Klinik IV, Ludwig-Maximilians-University Munich, Munich, Germany; ^7^School of Laboratory Medicine and Medical Sciences, University of KwaZulu-Natal, Durban, South Africa; ^8^Department of Tropical and Infectious Diseases, Institute of Primate Research, Nairobi, Kenya; ^9^Department of Infection and Immunity, University College London, London, United Kingdom; ^10^Department of Infection and Immunity, University College London, London, United Kingdom; ^11^Nuffield Department of Medicine, University of Oxford, Oxford, United Kingdom

**Keywords:** T cell, T cell receptor, cytomegalovirus, HLA-B^*^44:03, repertoire, sequencing

## Abstract

Lack of disease during chronic human cytomegalovirus (CMV) infection depends on the maintenance of a high-frequency CMV-specific T cell response. The composition of the T cell receptor (TCR) repertoire underlying this response remains poorly characterised, especially within African populations in which CMV is endemic from infancy. Here we focus on the immunodominant CD8+ T cell response to the immediate-early 2 (IE-2)-derived epitope NEGVKAAW (NW8) restricted by HLA-B^*^44:03, a highly prevalent response in African populations, which in some subjects represents >10% of the circulating CD8+ T cells. Using pMHC multimer staining and sorting of NW8-specific T cells, the TCR repertoire raised against NW8 was characterised here using high-throughput sequencing in 20 HLA-B^*^44:03 subjects. We found that the CD8+ T cell repertoire raised in response to NW8 was highly skewed and featured preferential use of a restricted set of V and J gene segments. Furthermore, as often seen in immunity against ancient viruses like CMV and Epstein-Barr virus (EBV), the response was strongly dominated by identical TCR sequences shared by multiple individuals, or “public” TCRs. Finally, we describe a pair “superdominant” TCR clonotypes, which were germline or nearly germline-encoded and produced at remarkably high frequencies in certain individuals, with a single CMV-specific clonotype representing up to 17% of all CD8+ T cells. Given the magnitude of the NW8 response, we propose that this major skewing of CMV-specific immunity leads to massive perturbations in the overall TCR repertoire in HLA-B^*^44:03 individuals.

## Introduction

Human cytomegalovirus (CMV) is a highly prevalent β-herpesvirus (1, 2). Although CMV infection can lead to severe clinical complications in immunocompromised patients such as in the setting of AIDS (3–5), in transplant patients on immunosuppressive regimens (6, 7) or following congenital infection (8), it is usually asymptomatic. CD8+ T cells play a central part of protection against CMV disease (9, 10). Adoptive transfer of CMV-specific CD8+ T cells into immunodeficient recipients effectively restores antigen-specific CD8+ T cell immunity, reduces CMV viraemia and prevents disease (9, 10). Conversely, lack of CD8+ T cell-mediated responses correlates with CMV replication and disease progression. The frequency of CMV-specific CD8+ T cells following natural infection is unusually high, constituting up to 10% of the CD8+ T cell memory compartment in peripheral blood in adults (11). These responses are maintained for decades and, in the case of certain specificities, may increase in frequency even further over time through a process termed “memory inflation” (12, 13).

Whether the size of the CMV-specific T cell response impacts the ability of the immune system to mount effective responses against other pathogens is unclear, but it is apparent that HIV disease progression is substantially more rapid in the case of HIV-CMV coinfection (14, 15). Age is also likely to be factor, and the contribution of CMV to immunosenescence and increased mortality from infectious disease in the elderly is still a topic of active debate (16, 17). Because the human T cell receptor (TCR) repertoire is finite and limited to ~25 million TCRs (18), it is possible that in individuals in whom 10% or more of CD8+ T cell responses are dedicated to control of CMV infection, the availability of optimal TCRs to control infections other than CMV might be constrained. This hypothesis remains untested, as high-throughput sequencing studies of TCR repertoires following CMV infection are relatively few, especially in populations where CMV is endemic from infancy (19, 20). In sub-Saharan Africa, most children become CMV-infected. in the first 3 months of life and CMV infection rates approach 90% in infants reaching 12 months of age (1, 21). Thus, understanding the molecular basis of CMV-specific T cellular responses has the potential to affect the lives of millions, in population groups that are particularly vulnerable.

We recently characterised an immunodominant CMV-specific response in African populations that is restricted by HLA-B^*^44:03 (22). This class I molecule is expressed in ~15% of African populations (23) and differs by a single amino acid from the closely related HLA-B^*^44:02 (24), the most prevalent HLA-B^*^44 subtype in Caucasian populations. Among individuals expressing HLA-B^*^44:03, an unusually high fraction of CD8+ T cells (up to 17%) is specific for the immediate-early-2 (IE-2_463−473_)-derived epitope NEGVKAAW (hereafter referred to as NW8). The disproportionate magnitude of the NW8-specific specific response in B^*^44:03+ individuals is likely to be underpinned by strong biases in the TCR repertoire, leading to preferential selection of NW8-specific CD8+ T cells against this HLA background, although this possibility has yet to be investigated.

Using high-throughput sequencing, we sought to characterise the TCR repertoire of peptide-MHC tetramer-sorted NW8-specific CD8+ T cells from 20 HLA-B^*^44:03+ CMV-infected individuals. We found that the TCR repertoire of NW8-specific CD8+ T cells displayed limited clonal diversity and usage of a restricted set of V and J genes. The response was characterised by the presence of identical or “public” TCRs in multiple unrelated individuals. Public TCR sequences were often dominant within an individual and characterised by central amino acid motifs which were found to be either entirely germline-encoded (in the case of TCR-α chains), or near germline (β chains). We conclude that the high-magnitude NW8 response causes a major perturbation of the TCR repertoire, with disproportionate expansion of germline clonotypes that are highly shared amongst HLA-B^*^44:03+ individuals.

## Materials and methods

### Study subjects

We studied 20 HIV-infected ART-naive adult subjects from a previously described cohort in Durban (South Africa) (25). The subjects studied had a mean age of 23 years (IQR, 21–31 years), a mean absolute CD4+ T cell count of 477/mm^3^ (IQR, 2025-107,500) and a median HIV viral load of 20,500 copies/ml (IQR 816–23,000). HIV viral load was measured using the Roche Amplicor version 1.5 assay according the manufacturer's instructions; CD4+ T cell counts were measured by flow cytometry. Samples utilised for global TCR repertoire sequencing from genomic DNA were obtained from subjects of similar ethnic background and also recruited in Durban, South Africa. Ethics approval, for both studies, was given by the KwaZulu-Natal Review Board (Durban cohort). All subjects provided written informed consent.

### Cell sorting

Peptide-MHC tetramers were generated as previously described (26). Cryopreserved PBMC (1 million per staining condition) from the recipients were stained with PE-conjugated or APC-conjugated peptide-MHC tetramers, anti-CD3 Pacific Orange (Invitrogen, UK), anti-CD8 V450 (BD Biosciences, Oxford, UK) antibodies and near-IR Live/Dead marker (Invitrogen, Paisley, UK). The samples were sorted on BD FACSAria (BD Biosciences, Paisley, UK) and tetramer+ CD8+ cells were collected in 350 μL of RLT lysis buffer (QIAGEN, Hilden, Germany).

### TCR sequencing

RNA extraction was carried out using the RNEasy Micro kit (Qiagen, Hilden, Germany) as previously described (27). Briefly, cDNA was synthesized using the 5′/3′ SMARTer kit (Takara Bio, Paris, France) according to the manufacturer's instructions. The SMARTer approach used a Murine Moloney Leukaemia Virus (MMLV) reverse transcriptase, a 3′ oligo-dT primer and a 5′ oligonucleotide to generate cDNA templates flanked by a known, universal anchor sequence. A reverse primer specific for the TCR-α or the TCR-β constant region (CαR1 5′ CCATAGACCTCATGTCTAGCACAG-3′ or CβR1 5′-GAGACCCTCAGGCGGCTGCTC-3′, Eurofins Genomics, Ebersberg, Germany) was then used together with an anchor-specific forward primer (Takara Bio, Paris, France) in the following PCR reaction: 2.5 μL template cDNA, 0.25 μL High Fidelity Phusion Taq polymerase, 10 μL 5x Phusion buffer, 0.5 μL DMSO (all from Thermo Fisher Scientific, UK), 1 μL dNTP (50 mM each, Life Technologies, Paisley, UK), 1 μL of each primer (10 μM), and nuclease-free water for a final reaction volume of 50 μL. Subsequently, 2.5 μL of the first PCR products were used to set up a nested PCR as above, using nested primers (CαR2 5′-GGTGAATAGGCAGACAGACTTGTC-3′ or CβR2 5′-TGTGTGGCCAGGCACACCAGTGTG-3′, Eurofins Genomics, Ebersberg, Germany and anchor-specific primer from Takara Bio, Paris, France). For both PCR reactions, cycling conditions were as follows: 5 min at 94°C, 30 cycles of 30 s at 94°C, 30 s at 63°C, 90 s at 72°C, and a final 10 min at 72°C. The final PCR products were loaded on a 1% agarose gel and purified with the QIAEX II gel extraction kit (Qiagen, Hilden, Germany). Purified products were barcoded, pooled and sequenced on an Illumina MiSeq instrument using the MiSeq v2 reagent kit (Illumina, Cambridge, UK).

TCR sequencing from genomic samples from whole blood was carried out by Adaptive Biotechnologies, using the Immunoseq platform,as previously described (28).

### TCR sequence analysis

Analysis of the raw TCR sequences was performed using MiXCR (29). MiXCR employs a built-in library of reference germline V, D, J, and C gene loci from the ImMunoGeneTics (IMGT) database (imgt.org). The IMGT nomenclature for TCR gene segments was used throughout the study. All aligned, in-frame, antigen-specific TCR clonotypes are available in the VDJDB repository at vdjdb.cdr3.net (30). By convention, the TCR third complementarity-determining region (CDR3) is written from the cardinal Cys residue to the conserved, J-encoded Phe residue.

### Statistical analysis

#### Repertoire clonality (evenness)

TCR repertoire clonality is given by the Shannon evenness index (*J*′):

$$J^{\prime }=\frac{H^{\prime }}{ln(n)}\;H^{\prime }=\sum_{i=1}^{n}p_{i}ln(pi)$$

Where *p*_*i*_ is the frequency of the *i*^*th*^ clonotype in a population of *n* clonotypes.

*J*′ is undefined for monoclonal samples. Low J′ values approaching 0 indicate minimal evenness as seen after clonal expansion of antigen-specific species. The maximal *J*′ value is 1, when all clonotypes have equal frequencies, i.e., the population is perfectly even.

#### Repertoire compositional similarity

Compositional similarity between TCR repertoires were assessed using the Morisita-Horn similarity index (*C*_*MH*_):

$$C_{MH}=\frac{2\sum_{i=\;1}^{c}f_{i}g_{i}}{\sum_{i=\;1}^{c}\left(f_{i}^{2}+g_{i}^{2}\right)}$$

where *f*_*i*_ = *n1*_*i*_*/N*_1_ *and g*_*i*_ = *n*_2i_*/N*_2_*, n*_1i_ and *n*_2i_ are the number of copies of the *i*^*th*^ clonotype in samples 1 and 2, and *N*_1_ and *N*_2_ are the total number of TCRs in samples 1 and 2, respectively. The summations in the numerator and the denominator are over the unique clonotypes (c) in both samples. The similarity indices range in value from 0 (minimal similarity; the samples are entirely different) to 1 (maximal similarity; the samples are identical). The *C*_*MH*_ similarity index accounts for both the number of common clonotypes and the distribution of clone sizes and is sensitive to the clone sizes of the dominant clonotypes. *C*_*MH*_ similarity calculations were performed using the “numpy” package in Python.

#### Statistical and graphical analysis

All pairwise statistical tests were performed in Prism v7.0 (GraphPad, San Diego, USA) unless stated otherwise. Strength of association between two variables was analysed by Spearman's rank test. *P* values < 0.05 were considered significant.

## Results

### NW8-specific T cells display limited TCR diversity

We sequenced the TCR-α and TCR-β chain repertoires of NW8-specific CD8+ T cells sorted from 20 HLA-B^*^44:03+ individuals from Durban, South Africa. The mean size of the tetramer+ population was 3.88% of CD3+CD8+ T cells (IQR 0.36-6.05) and the mean number of cells sorted for sequencing was 10,169 cells (IQR 327-11,791) (Supplementary Table 1 and Supplementary Figure 1). In total, 1,750,000 reads from TCR-α chain samples and 700,000 from TCR-β chains were generated using the Illumina MiSeq platform from these samples. This translated into 335,891 functional, in-frame TCR-α chain sequences and 91,154 TCR-β chains.

**Figure 1:**
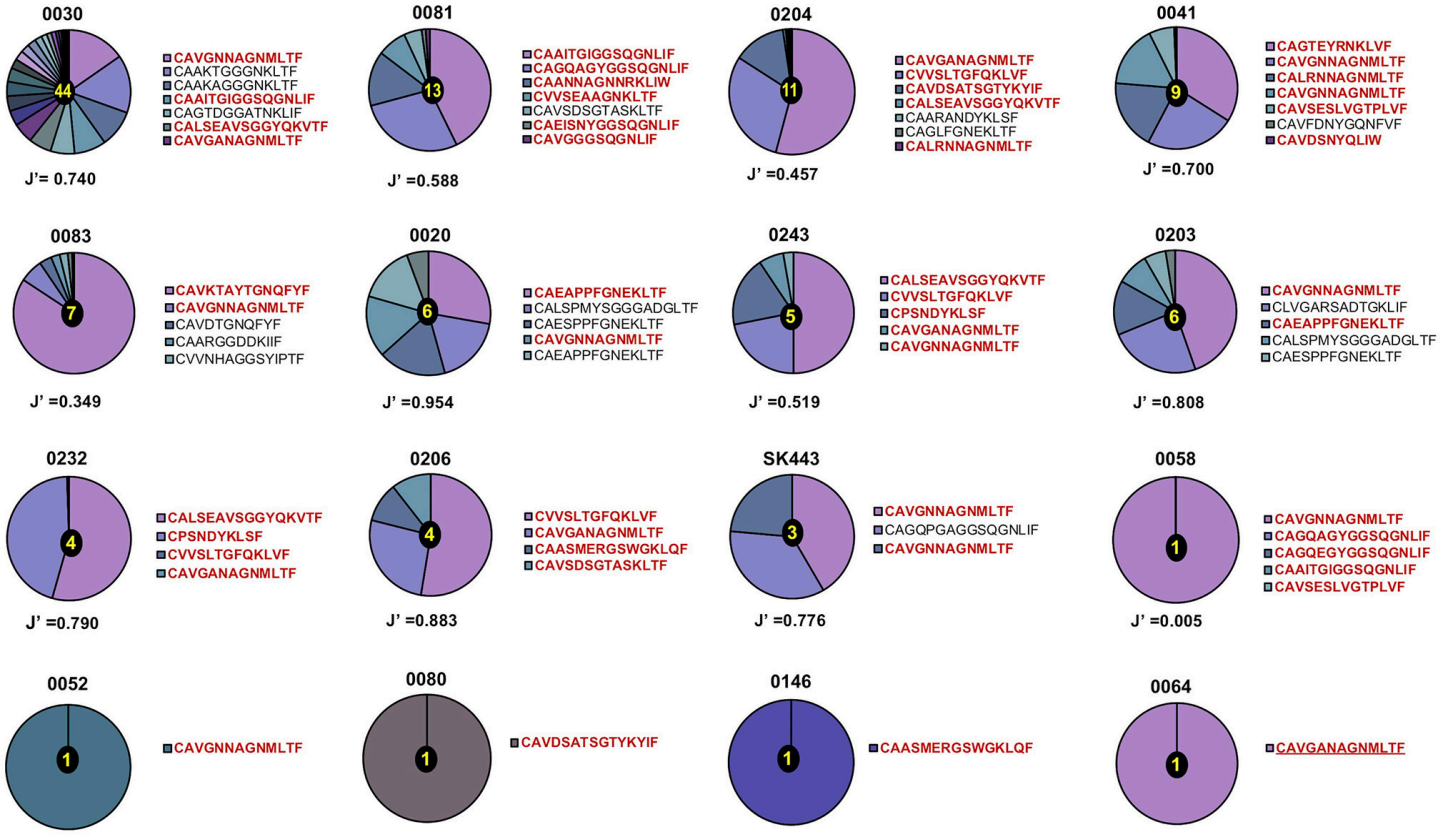
Distribution of TCR-α chain clonotypes in HLA-B^*^44:03/NW8-specific CD8+ T cells. The frequency of TCR-α chain amino acid sequences obtained from (*n* = 16) CMV-infected individuals is shown as pie charts. Frequencies of individual clonotypes are calculated as a percentage of aligned sequencing reads. Amino acid sequences of CDR3 loops are shown on the right of each pie, with public CDR3 clonotypes in bold red. Patient IDs are shown above each pie chart. Shannon's evenness index (J′) is indicated under each pie chart. The number in the centre of each pie indicates the total number of clonotypes seen in the corresponding patient. Colours are assigned randomly and do not correspond to a fixed a sequence.

**Figure 2:**
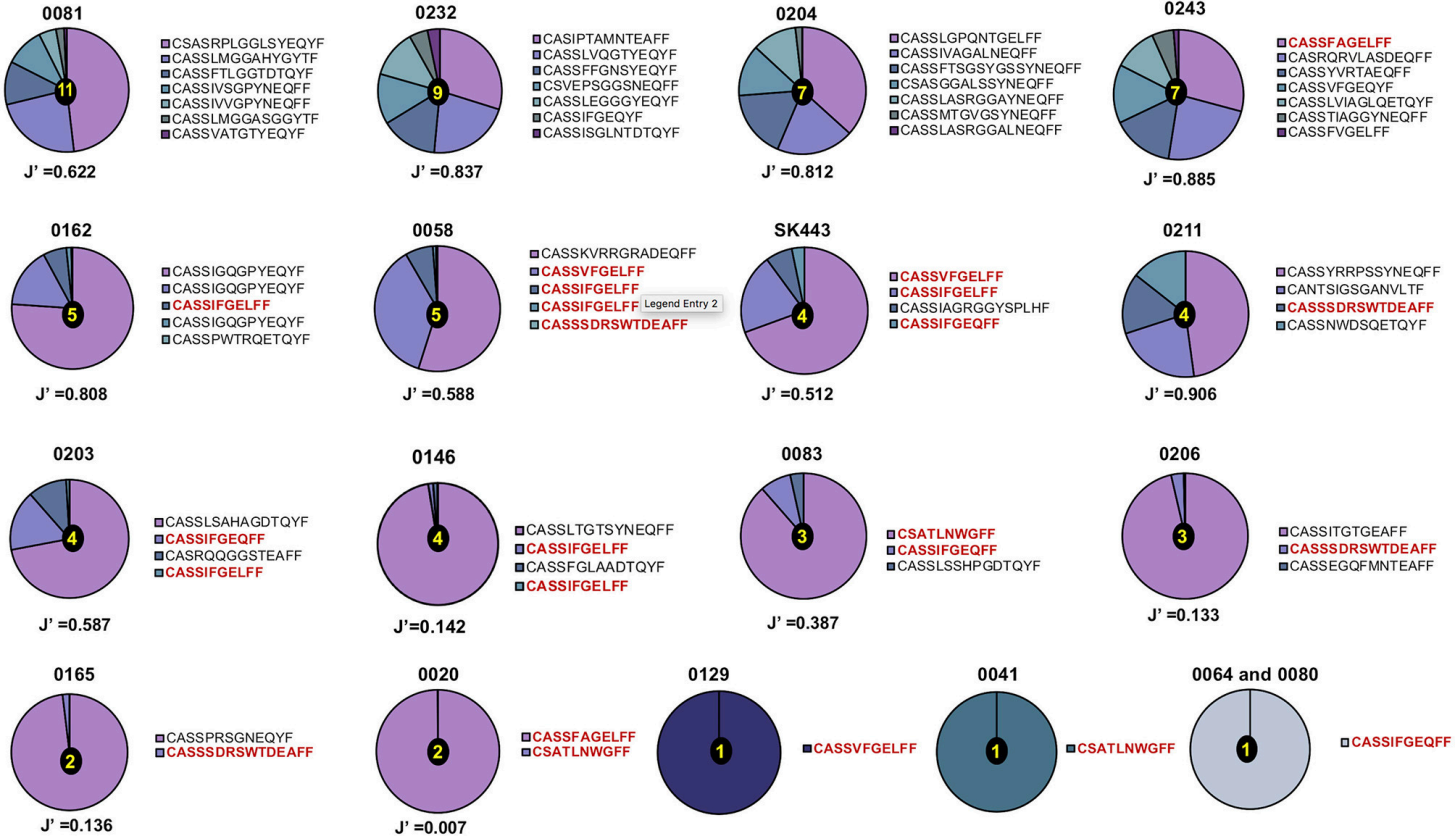
Distribution of TCR-β chain clonotypes in HLA-B^*^44:03/NW8-specific CD8+ T cells. The frequency distribution of TCR-β chain amino acid sequences obtained from (*n* = 18) CMV-infected individuals is shown as pie charts. Frequencies of individual clonotypes are calculated as a percentage of aligned sequencing reads. Amino acid sequences of CDR3 loops are shown on the right of each pie, with public CDR3 clonotypes in bold red. Patient IDs are shown above each pie chart. Shannon's evenness index (J′) is shown under each pie chart. The number in the centre of each pie indicates the total number of clonotypes seen in the corresponding patient. Colours are assigned randomly and do not correspond to a fixed a sequence.

A total of 53 TCR-α chain clonotypes were identified in 16 individuals and 51 TCR-β clonotypes in 18 individuals (Figures 1, 2). TCR repertoire richness, as measured by the number of clonotypes per patient varied widely across the cohort. This was particularly apparent in the case of TCR-α chains, for which the number of clonotypes ranged from 1 to 42. However, TCR-β chain samples were more homogeneous in size and the total numbers of TCR-β clonotypes only varied between 1 and 11. Although intuitively this could be attributed to variation in the number of sorted cells, the number of clonotypes did not correlate with the size of the tetramer+ population or with the number of cells sequenced (Supplementary Table 1). TCR clonality was evaluated using the Shannon evenness index (J′). J′ is undefined (approaching zero) when the sample is monoclonal, low when the distribution of clonotypes is uneven, and 1 when all clonotypes have the same frequency (See Materials and Methods). Four out of 16 TCR-α and five out of 18 TCR-β repertoires were strictly monoclonal (Figures 1, 2). Of particular interest was patient 0064 whose TCR-α and TCR-β repertoires were both monoclonal, which by default indicated that the NW8-specific response in this individual comprised of only one TCR. Other repertoires showed evidence of preferential expansion of a limited set of clonotypes, as suggested by low J′ values. To confirm this extreme oligoclonality was the result of bona fide antigen-specific expansion and not methodological bias, TCR-α and TCR-β chain samples were amplified using an independent primer set, cloned into a plasmid vector and sequenced. Using this alternative approach, we found that the clonotypic hierarchy was preserved, confirming the limited TCR diversity in NW8-specific response as genuine (Supplementary Figure 2). Thus, both the TCR-α and the TCR-β repertoires of NW8-specific CD8+ T cells displayed evidence of clonal expansion, which in some cases led to dominance of a single clone, or very few clones at best.

### The NW8-specific CD8+ T cell repertoire is characterised by restricted V and J segment usage

**Figure 3:**
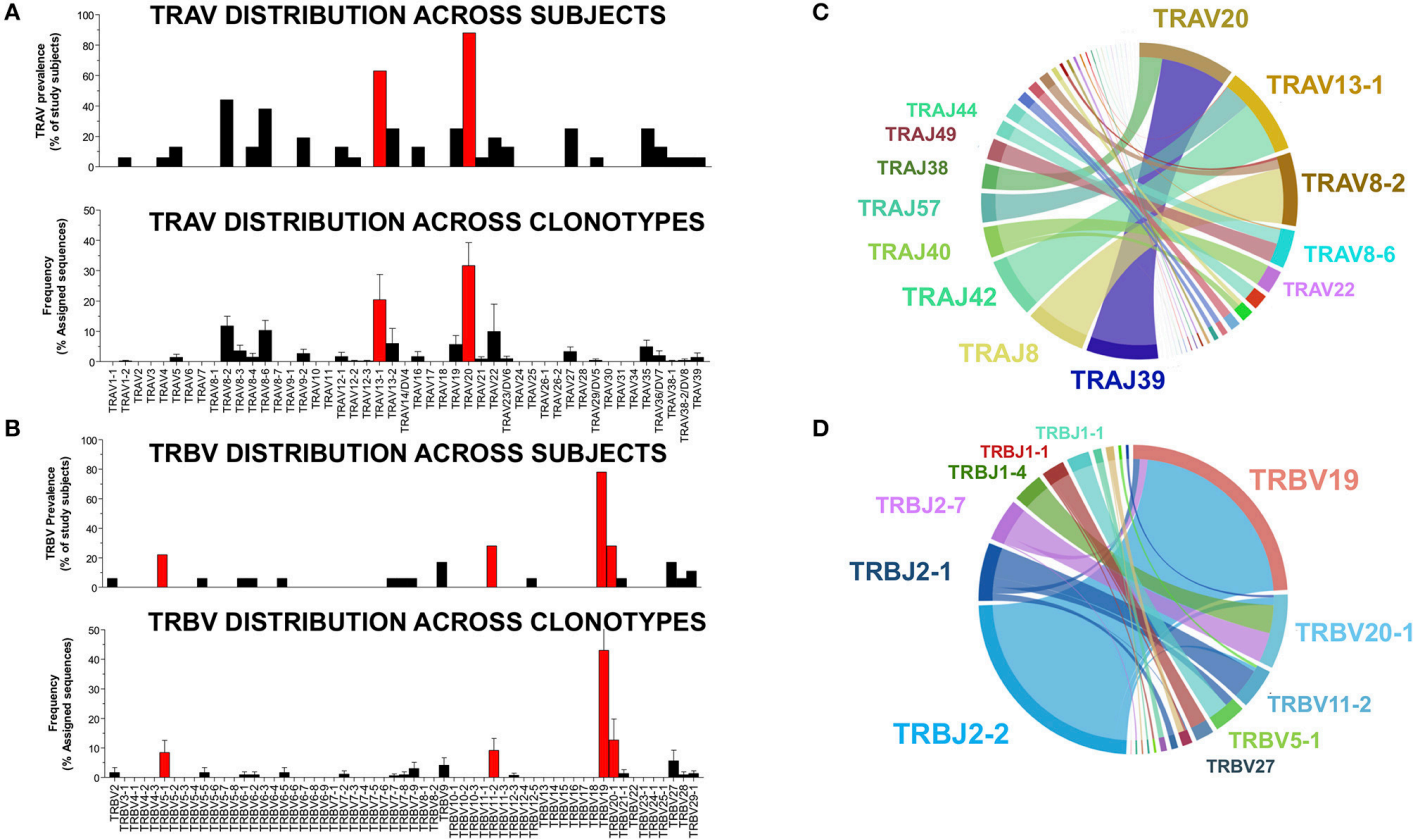
V and J gene usage in TCR-α and –β chain repertoires of HLA-B^*^44:03/NW8-specific CD8+ T cells. The distribution of TRAV genes **(A)** is shown (*n* = 16) individuals (top panel). The frequency of TRAV genes found in NW8-tetramer-sorted cells is expressed as a frequency of all assigned sequences (bottom panel). Individual bars indicate the mean frequency and standard error of the mean (SEM). Red bars indicate TRAV genes encoding TCR-α chains which are public. The distribution of TRBV genes **(B)** is shown (*n* = 18) individuals (top panel). The frequency of TRBV genes found in NW8-tetramer-sorted cells is expressed as a frequency of all assigned sequences (bottom panel). Individual bars indicate the mean frequency of a V gene and SEM. Red bars indicate TRBV genes encoding TCR-β chains which are public. TRAV-TRAJ co-occurrence maps are shown in **(C)**, representative of 16 TCR-α repertoires. TRBV-TRBJ co-occurrence maps are shown in **(D)**, representative of 18 TCR-β repertoires. The size of the arcs is proportional to TRAV, TRBV, TRAJ, or TRBJ frequency, the area joining any V-J pair is proportional to the frequency of that V-J pair. Co-occurrence maps were generated by VDJTOOLS as described in (30).

TCRs are generated early in T cell ontogeny by recombination of V (D, in the case of TCR-β) and J gene segments (31). Antigen-driven selection of TCR clonotypes often leads to skewed distributions of V and J genes (32–35). Because this phenomenon is particularly apparent in the setting of human viral infection, we sought to determine whether NW8-driven clonal selection would also lead to a narrowing of V and J gene usage. We looked at the distribution of TRAV and TRBV genes, first across individuals, then as a proportion of all sequences obtained in the study. NW8-specific CD8+ T cells indeed displayed strongly skewed gene usage (Figure 3). Out of the 16 individuals sequenced, 14 (87%) harboured at least one TCR encoded by the TRAV20 gene segment. Moreover, almost a third (32%) of all TCR-α chains were encoded by the TRAV20 gene segment (Figure 3A). Similarly, the TRBV19 gene segment was found in 15 out of the 18 individuals sequenced (83%), and 43% of all TCR-β sequences were encoded by TRBV19 (Figure 3B). Accordingly, certain TRAV-TRAJ gene combinations were more frequent than others. Among TCR-α chain repertoires, the TRAV20-TRAJ39 pair was the most frequent (Figure 3C). TCR-β sequences showed an even more striking bias for TRBV19 pairing with TRBJ2-2, 2-1 and 2-7 (Figure 3D). Thus, the NW8-specific CD8+ T cell repertoire is characterised by restricted V and J segment usage. Altogether, the preferential selection of the TRAV20 and TRBV19 genes across individuals with a small set of TRAJ/TRBJ (respectively) is evidence for antigen-driven selection of public clonotypes (further discussed in the next section).

### High-frequency TCR clonotypes are highly shared amongst HLA-B^*^44:03+, CMV-infected individuals

**Figure 4:**
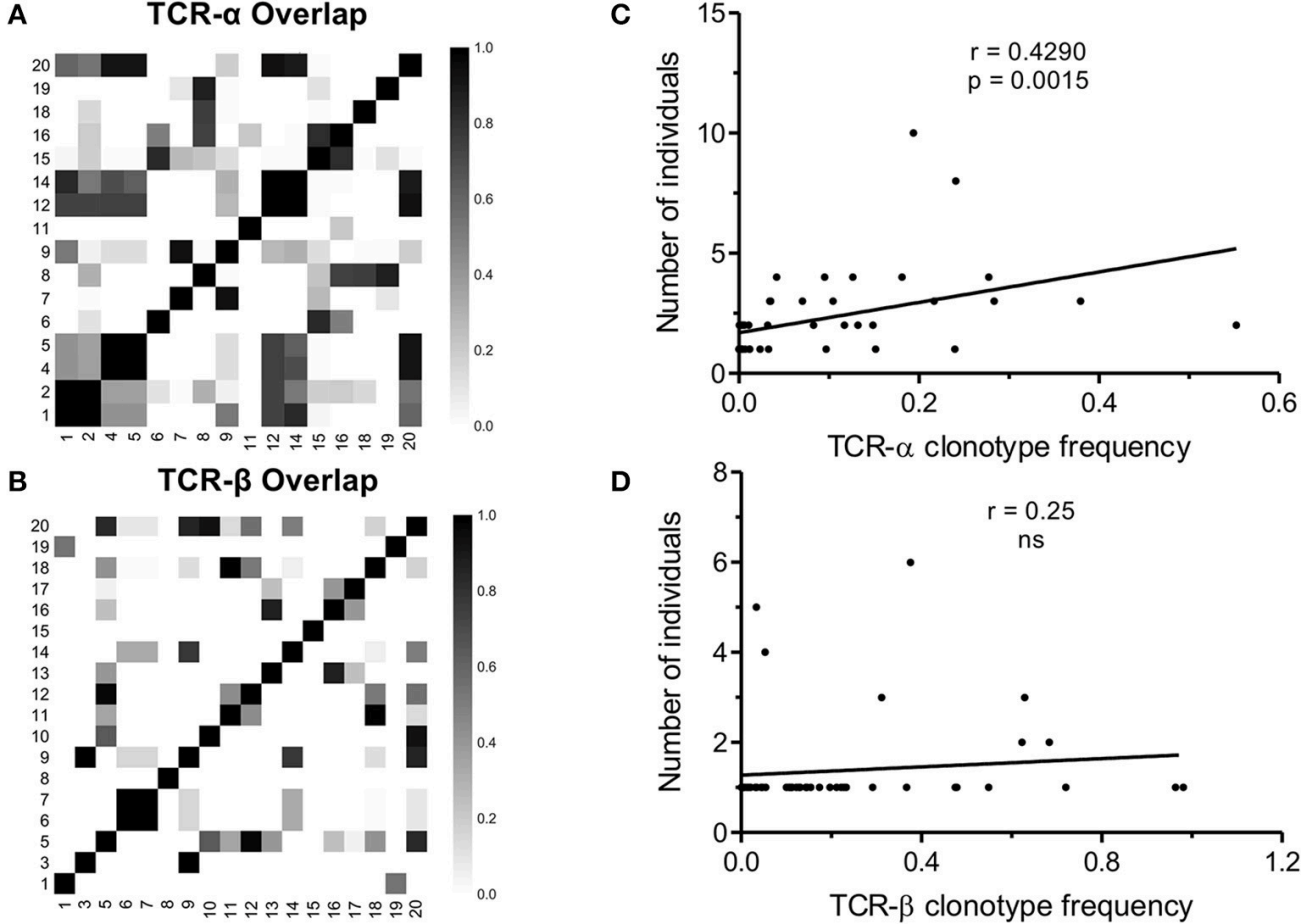
TCR sharing amongst NW8-specific CD8+ T cells from CMV-infected individuals. Compositional similarity amongst **(A)** TCR-α and **(B)** TCR-β chains was assessed using the Morisita-Horn (C_MH_) index and illustrated as a heat map. Patient numbers are shown along the x and y-axes. C_MH_ values range from 0 (no overlap) to 1 (perfect overlap). The average frequency of a given clonotype is plotted against the number of individuals sharing that clonotype, for **(C)** TCR-α chains (*n* = 53) and **(D)** TCR-β chains (*n* = 51). Spearman's rho and *p*-values are shown above each graph.

Viral infection such as CMV often leads to the selection of predictable repertoires with extensive sharing of TCR clonotypes (31, 36). As mentioned above, the selection of a narrow set of V and J genes pointed to the public nature of the NW8. To test whether NW8-specific CD8+ T cells were indeed public, we used the Morisita-Horn similarity-index (C_MH_) to perform pairwise comparisons between every combination of two donors, as a measure of compositional similarity, or overlap (Figure 4). C_MH_ values lie between 0 (non-overlapping populations) and 1 (identical populations). The C_MH_ value for every combination of two donors, representing a total of 120 pairwise combinations for the TCR-α chain and 153 for TCR-β (see Materials and Methods). The mean C_MH_ value of the TCR-α chain repertoire was 0.20 ± 0.02 (Figure 4A), indicating that on average, the patients in this study shared a fifth of their NW8-specific, TCR-α repertoire. Despite strong selection for TRBV19+ clonotypes, the mean C_MH_ of TCR-β repertoires was only 0.09 ± 0.10 (Figure 4B). The lower C_MH_ in TCR-β chain repertoires is likely to merely reflect the inclusion of a D segment in TCR-β transcripts, leading to more junctional diversity, compared to TCR-α. Nonetheless, multiple public TCR chains were identified, both in TCR-α and TCR-β repertoires (Figures 1, 2, 4).

Out of 53 TCR-α chain clonotypes, 32 were public. Two related clonotypes, CAVGNNAGNMLTF and CAVGANAGNMLTF, both TRAV20/TRAJ39 as anticipated and differing by just a single amino acid, were the two most abundant of all clones sequenced (30.4 and 3.7%, respectively). These clones were also the most extensively shared. CAVGNNAGNMLTF was found in 12/16 (75%) of subjects studied and CAVGANAGNMLTF in 7/16 (44%). To confirm this extreme bias, TCR-α chains were sequenced using the Immunoseq platform from whole blood genomic DNA of 26 individuals of the (28) same ethnicity, four of which were HLA-B^*^44:03+ (Supplementary Figure 3). The frequency of the CAVGANAGMLTF and CAVGNNAGMLTF clonotypes cumulated to 3.3, 1.1, and 2.3% of total productive TCRs (derived from both CD4+ and CD8+ T cells), in three of the four HLA-B^*^44:03+ subjects. By contrast, in HLA-B^*^44:03-negative subjects, the frequency of these clonotypes did not exceed 0.01%. This demonstrates, using a different methodology, that the superdominant clonotypes CAVGANAGMLTF and CAVGNNAGMLTF are highly enriched in HLA-B^*^44:03+ individuals, and expanded to comparably high frequencies as observed by tetramer staining.

Similarly, the most abundant public TCR-β clonotypes, CASSIFGEQFF and CASSIFGELFF (TRBV19/TRBJ2-1 and TRBV19/TRBJ2-2, respectively), were shared by 7/18 (39%) of individuals studied. Consistent with previous observations (37), the higher the frequency of a given clonotype, the greater the likelihood that this would be a public TCR sequence. This correlation between clonotype frequency and the degree of publicity was statistically significant for TCR-α chains (Spearman's *r* = 0.4290, *p* = 0.0015; Figure 4C) but not for TCR-β chains (Figure 4D). This suggests that the previously described relationship between clonal abundance and publicity is weak in the case of the NW8 response and that other factors contribute to “superdominance” of certain TCR clonotypes in HLA-B^*^44:03+ individuals.

The public amino acid sequences were often encoded by more than one distinct nucleotide sequence (Supplementary Tables 2, 3 and Supplementary Figure 4). For example, the most prevalent public TCR-β clonotype, CASSIFGEQFF, was encoded by four different nucleotide sequences. Similarly, the most frequently occurring TCR-α public clonotype, CAVGNNAGNMLTF, was also encoded by four different nucleotide sequences. Clonotypes that occurred in a greater number of individuals tended to also be encoded by a greater number of distinct nucleotide sequences. This is often referred to as convergent recombination, whereby redundancy in the genetic code leads to generation of TCR clonotypes with degenerate nucleotide sequences at high frequency (38, 39). For TCR-β chains, this association was statistically significant (Spearman's *r* = 0.798, *p* < 0.0001, Supplementary Figure 4).

Overall the high prevalence of public TCRs in NW8-specific CD8+ T cells, that can be encoded by several nucleotide sequences, is further evidence of the strong selection for particular TCR amino acid sequences by the HLA-B^*^44:03-NW8 peptide-MHC complex. The public nature of the NW8 response is likely to be underpinned by the conservation of structural features allowing the selection of “superdominant,” highly prevalent TCR chains against this MHC background.

### Public CDR3 motifs are strictly conserved amongst HLA-B^*^44:03+, CMV-infected individuals

**Figure 5:**
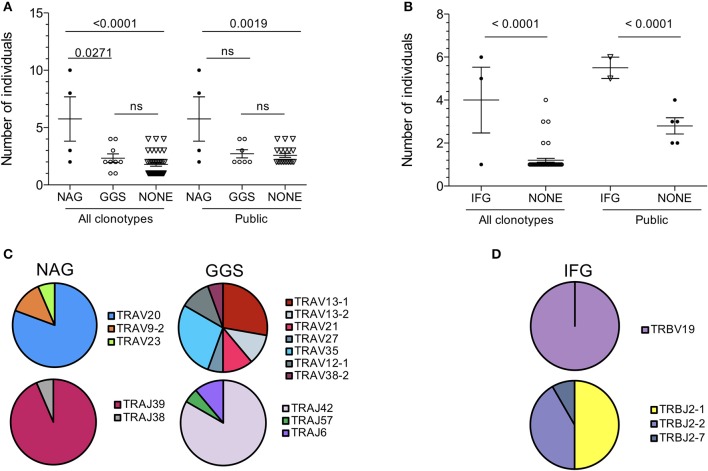
Amino acid motif conservation in public clonotypes from HLA-B^*^44:03/NW8-specific CD8+ T cells. **(A)** The number of individuals sharing NAG or GGS-containing TCR-α chains is shown. **(B)** The number of individuals sharing IFG-containing TCR-β chains is shown. **(C)** TRAV and TRAJ gene usage in NAG or GGS-containing TCR-α chains is illustrated as pie charts. Frequencies are calculated as fraction of NAG or GGS- containing amino acid sequences. **(D)** TRBV and TRBJ gene usage in IFG-containing TCR-β chains is illustrated as pie charts. Frequencies are calculated as fraction of IFG-containing amino acid sequences. Horizontal bars represent the mean and standard of the mean. Statistical significance is shown where *p* < 0.05 (ns = not significant). Pie charts illustrate the frequency of a V or J gene. Colours are assigned randomly and do not correspond to a fixed a sequence.

TCR repertoire bias often includes the conservation of amino acid motifs within CDR3 loops (33, 34, 40, 41). Here we observed that several TCR-α chain clonotypes contained the sequence motif Asn-Ala-Gly (referred to as NAG thereafter) or Gly-Gly-Ser (GGS). TCR-α chains with the NAG motif were shared across more individuals than those with no motif (*p* < 0.0001) or with the GGS motif (*p* = 0.0271; Figure 5A). This also held true for TCR-β chains containing the Ile-Phe-Gly (IFG) motif (Figure 5B). All TCR chains with a conserved motif were public. Conversely, private TCR chains displayed no apparent motif. The NAG motif was entirely encoded by the TRAJ region, specifically the TRAJ39 segment in 93.5% of clonotypes. Similarly, the GGS motif was largely encoded by TRAJ42. Strikingly, V gene usage was not conserved. NAG clonotypes were encoded by three different TRAV genes and GGS by as many as seven TRAV genes (Figure 5C). The use of several TRAV genes likely excludes the possibility of a germline-encoded interaction between TCR-α CDR1 and −2 loops with MHC, as has been observed with other epitopes (42, 43), and suggests a more prominent role for CDR3 loops in NW8 recognition.

TCR-β chains clonotypes with the IFG motif were strictly encoded by TRBV19, while TRBJ usage was less conserved (Figure 5D). Again, all IFG motif-containing chains were public. The central phenylalanine residue at position 6 was not germline-encoded in all cases and resulted from two different codons. The conservation of a non-germline residue again argues for structurally-imposed, antigen-driven selection of clonotypes bearing this amino acid at this position. Overall, we conclude that TCR publicity in the NW8-specific response is governed by structural constraints selecting for germline or near-germline encoded CDR3 loops.

## Discussion

In this study we sought to characterise the TCR repertoire of CD8+ T cells responding to an immunodominant CMV epitope in HLA B^*^44:03+ individuals. Using high-throughput sequencing, we dissected the NW8-specific TCR-α and –β chain repertoires of 20 CMV-infected subjects. These repertoires were found to be highly skewed, as indicated by preferential use of V and J genes, extensive TCR sharing and conservation of CDR3 motifs. TCR diversity and the specificity it underlies are the distinguishing feature of αβ T cells. Diversity in T cell responses has long been thought of as a strict requirement for the provision of protective immunity and full immune coverage. Nonetheless, it is now well established that human viral infections can give rise to narrow and skewed repertoires, often associated with TCR sharing in different individuals. Thus, the repertoire is often shaped toward a single or focused solution for the recognition of peptide-HLA complexes. Reports of TCR publicity in immune responses have accumulated in the literature, such that TCR sharing across individuals can now almost be taken as a rule, rather than the exception (36, 41, 44, 45).

The occurrence of public TCRs is nearly 10^4^ times more likely than expected if all rearrangements were equiprobable, irrespective of HLA type (18). This is because public TCRs have distinct features, such as limited length and P/N diversification, which allows them to be generated frequently by the recombination machinery. A TCR sequence will have a higher likelihood of being produced if it is encoded by a nucleotide sequence with few additions and deletions. Redundancy in the genetic code also explains why TCR chains containing residues encoded by multiple codons (such as Gly or Ala) are very commonly produced and can occur at high frequency (38, 39). TCR production frequency has already been described as an important determinant of TCR sharing (37). Here, the public chains described in this study were nearly entirely germline-encoded or involved very limited changes at the CDR3 junction. This was effectively mirrored by the conservation of amino acid motifs. The TCR-α chain “NAG” motif described here was found to be entirely encoded by the J segment, and the “IFG” motif found in TCR-β chains only contained one added amino acid. The germline nature of these public chains again highlights the importance of TCR generation efficiency. In the case of NAG TCR-α chains, the conservation of the J segment, but not the V segment, excludes a potential role for germline CDR1 and CDR2 in recognition of HLA-B^*^44:03 (46). Rather, this emphasises the starring role of the CDR3 loop in antigen recognition and structural constraints favouring the selection of public clonotypes with these conserved motifs (32–34).

The other distinguishing feature of NW8-specific repertoires was their extremely limited diversity. In some individuals, TCR-α and TCR-β chain repertoires were even monoclonal. This was notably the case of patient 0064, with a tetramer response of 16.8% (equivalent to 31,480 sorted cells) composed of a single TCR, CAVGANAGMLTF-CASSIFGEQFF. This, to our knowledge, is the largest reported T cell response mounted by a single public T cell clone in humans, which initially led us to envisage a positive relationship between TCR publicity and the magnitude of the T cell response. However, we found that this was not the case, as the number of public TCRs did not correlate with the percentage of tetramer-positive cells or with the number of sorted cells. Instead, the frequency of a given clonotype positively correlated with the extent to which it is shared (i.e., the number of individuals harbouring that clonotype). Altogether, these results suggest that publicity in T cell responses does not necessarily equate to superior immunity. Rather, the dominance of public TCRs within an individual and across individuals can be explained by their high precursor frequency, or in other words, by a numerical competitive advantage (47).

The patient cohort recruited for this study were HIV-1 co-infected and receiving ART and it is well-established that such individuals have heightened CMV-specific T cell responses. Whether the expansion of superdominant clonotypes stems from dysregulated and heightened responses resulting from HIV-1 co-infection remains to be tested. It has been suggested that the increased frequency of CMV-specific T cells in ART patients may be due to abnormal responses to otherwise normal levels of subclinical CMV replication (48). Similarly, the disproportionate representation of superdominant clonotypes could be another manifestation of HIV-mediated immune dysregulation.

The unusual magnitude of the HLA-B^*^44:03-restricted NW8-specific CD8+ T cell response deserves comment. In most individuals, >3% of CD8+ T cells, and in one individual almost 1 in 5 CD8+ T cells (19%), are specific for this single response (22). Even by the standards of CMV-specific CD8+ T cell responses, which are renowned to be very large, this is an exceptional example. The progressive, prolonged expansion of these responses has been termed memory “inflation” (12, 49–51). It has been suggested that these accumulations of CMV-specific CD8+ T cells with age may be responsible for shortening of the human life-span as a result of excessive loss of naïve T cell numbers (52). If this is the case, it might be predicted that individuals with HLA-B^*^44:03 might be at particular risk, not only of reduced ability to counter infections in later life, but also to combat coinfections such as HIV which also appear to induce large T cell expansions (53).

How this so-called memory “inflation” is established upon infection remains unclear, although it is possible that certain pathogens, like CMV, would adapt to host immunity to abrogate the contraction phase of the immune response. The composition of the “inflated” memory pool both at the cellular and clonotypic levels has also remained elusive (54). Our results are consistent with the notion that, in HLA-B^*^44:03 individuals, the CD8+ T cell response to the NW8 epitope is associated with remarkably large clonotypic expansions reminiscent of memory inflation. In the absence of longitudinal TCR analysis, this hypothesis remains to be tested formally. Nonetheless, it is clear that this repertoire is a very limited repertoire of “superdominant clones” that in the extreme can occupy almost 20% of the CD8+ T cell pool. Whether this feature is typical of memory inflation in general, or specific to CMV, will have to be determined.

Intuitively, that highly prevalent pathogens such as CMV are best dealt with by TCRs which are highly prevalent across a population appears to be a beneficial trait. The *mhc* locus is the most gene-dense and the most polymorphic genetic complex known in jawed vertebrates, reflecting strong evolutionary pressure from highly mutable pathogens (55). Polymorphism at the *mhc* affects an individual's immune status by determining the collection of peptides made available to T cells during development and shaping of the TCR repertoire and also upon antigenic challenge (56). “Cat and mouse” evolution between the host immune system and invading pathogens is a well-documented phenomenon, particularly in the case of rapidly mutating pathogens such as retroviruses. By contrast, the high prevalence and genetic stability of herpesviruses such as CMV may lead to the conservation of a TCR genotype that is widely shared across the population as a result of frequent generation and selection of clonotypes which are germline or near-germline-encoded during T cell development (41, 57). Whether the “superdominant,” public TCRs identified in this study represent a built-in bank of receptors which can readily be mobilised against ubiquitous pathogens like herpesviruses remains to be determined. Understanding the molecular rules which govern how TCRs are generated and selected to seed the periphery will be crucial in our understanding of public T cell responses.

## Author contributions

The study was conceived by AS and PG and funded by grants to AS and PG. MA, AM, MS, JR, and PO did the experimental work that was supervised by TN, SB, HK, PM, AS, and PG. The data were analyzed by MA, AM, HK, AS, and PG. The manuscript was written by MA, AM, AL, HK, PM, AS, and PG.

### Conflict of interest statement

The authors declare that the research was conducted in the absence of any commercial or financial relationships that could be construed as a potential conflict of interest. The reviewer EH declared a past co-authorship with one of the authors AS to the handling editor.

## Supplementary material

Supplementary Figure 1 Tetramer-based sorting of CMV-specific CD8+ T cells. **(A)** tetramer+ populations are shown in flow cytometry plots, with HLA-B^*^44:03-SL9 (derived from HIV-p24) tetramer along the y-axis and HLA-B^*^44:03-NW8 tetramer along the x-axis. The plots fail to show double-positive cell populations, indicating specificity of staining. Patient identification number are shown in red within each plot. **(B)** FACS plots showing the gate boundaries set for sorting of CD8+HLA-B^*^44:03-NW8 tetramer+ cells (labelled as P2, orange). The gates were set on Live CD3+CD8+ cells. Patient identification number are shown in red within each plot.

Supplementary Figure 2 Sanger sequencing confirms clonal dominance in TCR-α and TCR-β chain repertoires. The frequency of TCR-α (top) and TCR-β chain (bottom) clonotypes is shown for four patients. Pie charts on the left-hand side represent data obtained using high-throughput sequencing, on the right-hand side Sanger sequencing.

Supplementary Figure 3 Superdominant clonotypes are readily identifiable in HLA-B^*^44:03-positive individuals. TCR-α chain sequencing was carried out on genomic DNA derived from whole blood, using the “ImmunoSeq platform” available at Adaptive Biotechnologies (28). The cumulative frequency of the superdominant clonotypes CAVGANAGMLTF and CAVGNNAGMLTF is shown for *n* = 26 individuals, of which four are HLA-B^*^44:03+ (red bars).

Supplementary Figure 4 Number of nucleotide sequences and TCR publicity. The number of nucleotide sequences encoding a given clonotype is plotted against the number of individuals sharing that clonotype, for **(A)** TCR-α chains and **(B)** TCR-β chains. Spearman's rho and *p*-values are shown above each graph.

Supplementary Table 1 Patient HLA-B type and size of tetramer-specific response. Four-digit HLA-B types are given for each patient, along with the size of the tetramer -specific response. Tetramer+ cells were gated on live CD3+CD8+ T cells.

Supplementary Table 2 Public TCR clonotypes are encoded by multiple nucleotide sequences. The nucleotide and amino acid sequence of superdominant public clonotypes is shown, together with their absolute read count and relative frequency.

Supplementary Table 3 TCR-α chain clonotypes are shared amongst HLA-B^*^44:03+ individuals and encoded by multiple nucleotide sequences. TCR-α chain sequencing from genomic DNA was carried out in three HLA-B^*^44:03+ individuals, using the Immunoseq platform (Adaptive Biotechnologies). Nucleotide and amino acid sequences are shown, together with their absolute count and relative frequency.
