# Supplementary figures and images for: Major TCR Repertoire Perturbation by Immunodominant HLA-B*44:03-Restricted CMV-Specific T Cells
Source: Front Immunol. 2018 Nov 14;9:2539. doi: 10.3389/fimmu.2018.02539 (PMC6246681; doi:10.3389/fimmu.2018.02539)

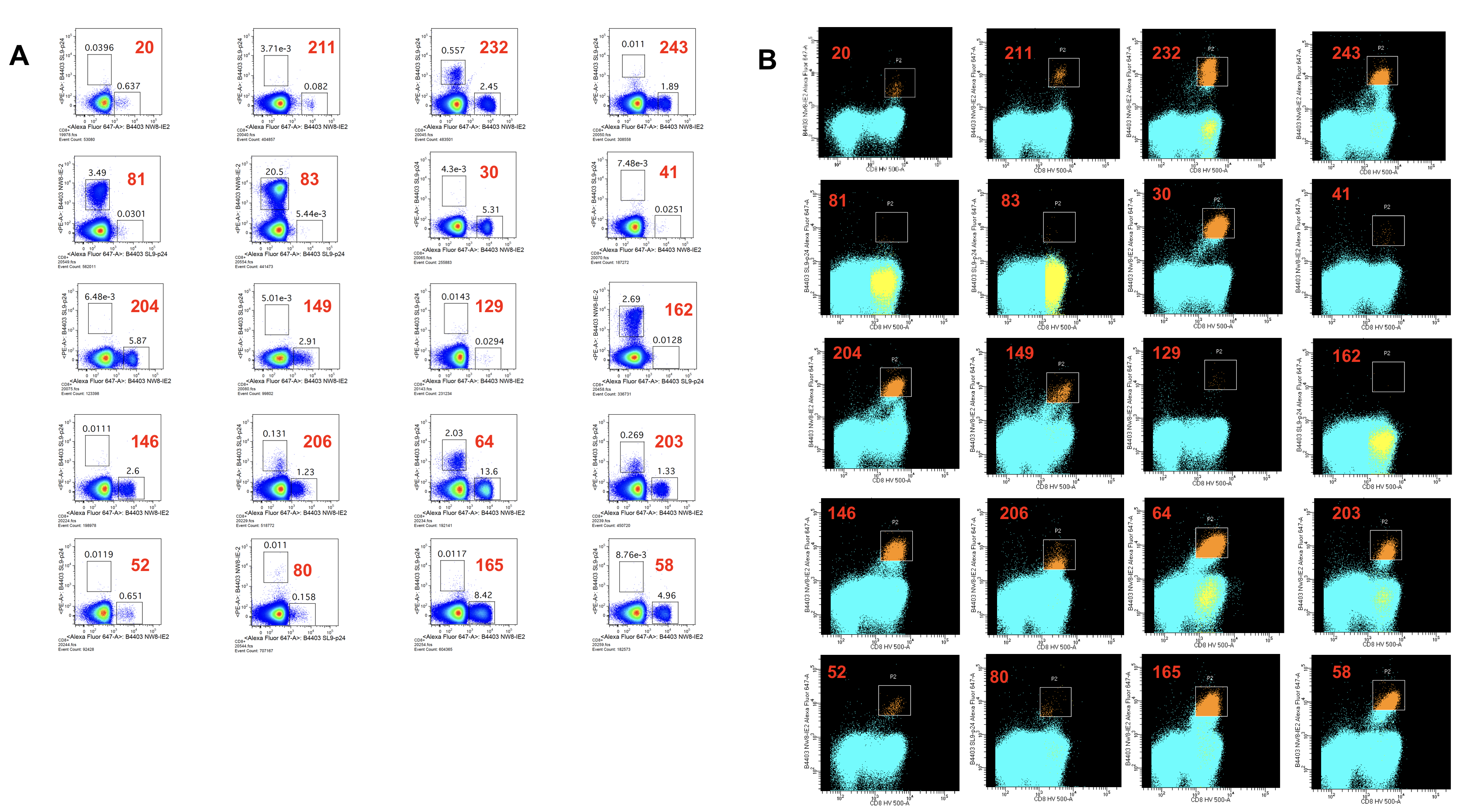

Supplement: Supplementary Figure 1 — Tetramer-based sorting of CMV-specific CD8+ T cells. (A) tetramer+ populations are shown in flow cytometry plots, with HLA-B*44:03-SL9 (derived from HIV-p24) tetramer along the y-axis and HLA-B*44:03-NW8 tetramer along the x-axis. The plots fail to show double-positive cell populations, indicating specificity of staining. Patient identification number are shown in red within each plot. (B) FACS plots showing the gate boundaries set for sorting of CD8+HLA-B*44:03-NW8 tetramer+ cells (labelled as P2, orange). The gates were set on Live CD3+CD8+ cells. Patient identification number are shown in red within each plot. [file Image_1.TIFF]

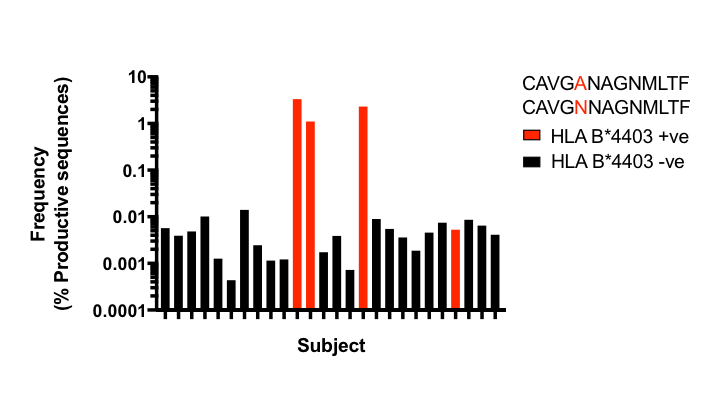

Supplement: Supplementary Figure 3 — Superdominant clonotypes are readily identifiable in HLA-B*44:03-positive individuals. TCR-α chain sequencing was carried out on genomic DNA derived from whole blood, using the “ImmunoSeq platform” available at Adaptive Biotechnologies (28). The cumulative frequency of the superdominant clonotypes CAVGANAGMLTF and CAVGNNAGMLTF is shown for n = 26 individuals, of which four are HLA-B*44:03+ (red bars). [file Image_3.TIFF]

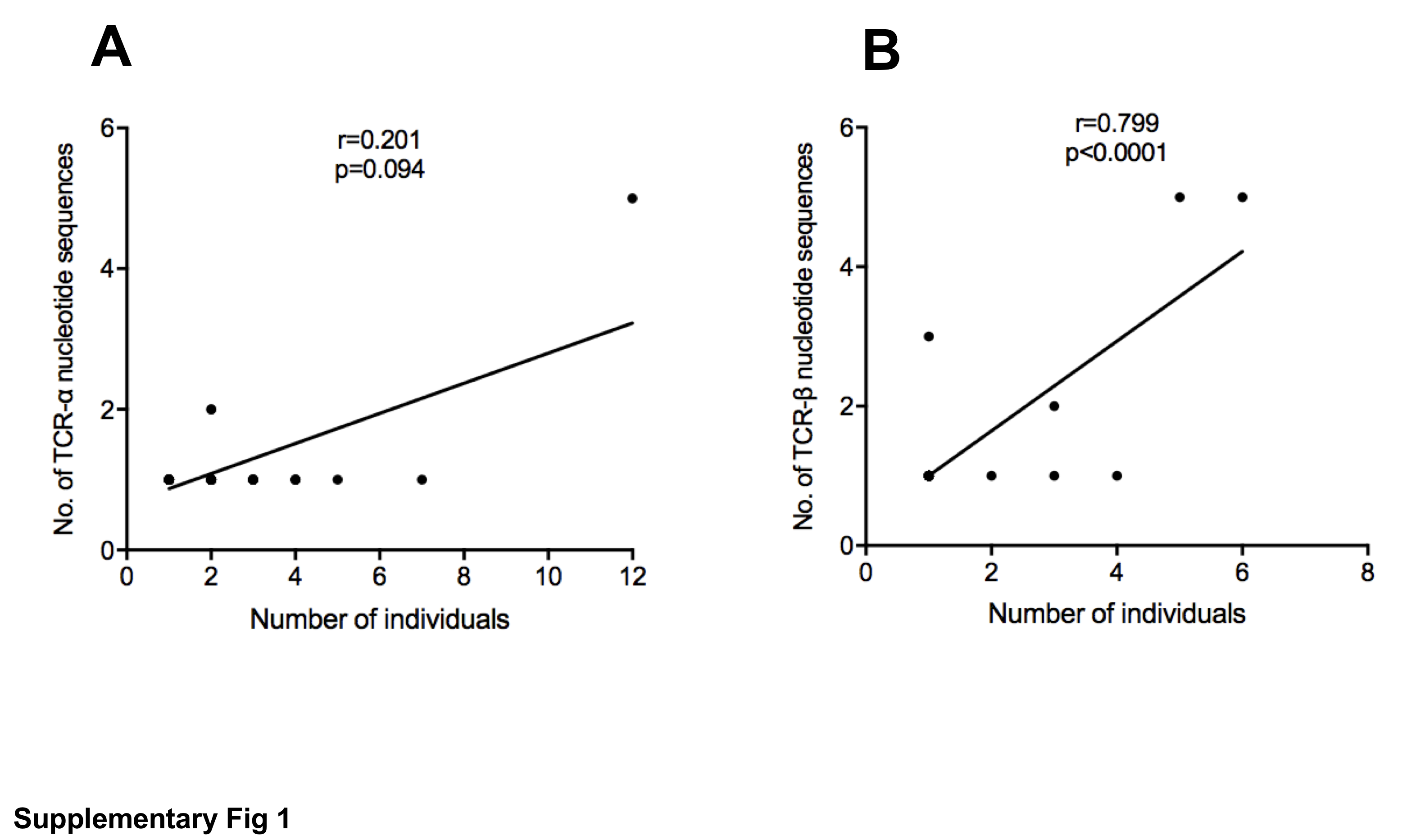

Supplement: Supplementary Figure 4 — Number of nucleotide sequences and TCR publicity. The number of nucleotide sequences encoding a given clonotype is plotted against the number of individuals sharing that clonotype, for (A) TCR-α chains and (B) TCR-β chains. Spearman's rho and p-values are shown above each graph. [file Image_4.TIFF]
