# Supplementary material for: Major TCR Repertoire Perturbation by Immunodominant HLA-B*44:03-Restricted CMV-Specific T Cells
Source: Front Immunol. 2018 Nov 14;9:2539. doi: 10.3389/fimmu.2018.02539 (PMC6246681; doi:10.3389/fimmu.2018.02539)
Supplement: Supplementary Table 1 — Patient HLA-B type and size of tetramer-specific response. Four-digit HLA-B types are given for each patient, along with the size of the tetramer -specific response. Tetramer+ cells were gated on live CD3+CD8+ T cells. [file Table_1.DOCX]

**Supplementary Table 1. Patient HLA-B type and size of tetramer-specific response.** Four-digit HLA-B types are given for each patient, along with the size of the tetramer -specific response. Tetramer+ cells were gated on live CD3+CD8+ T cells.

| Patient number | HLA-B | | %tetramer+ |
| --- | --- | --- | --- |
| 1 | 44:03 | 49:01 | 0.39 |
| 2 | 44:03 | 82:02 | 7.29 |
| 3 | 44:03 | 81:01 | 0.02 |
| 4 | 07:02 | 44:03 | 0.44 |
| 5 | 44:03 | 82:02 | 6.07 |
| 6 | 44:03 | 58:01 | 16.8 |
| 7 | 44:03 | 81:01 | 0.01 |
| 8 | 35:01 | 44:03 | 2.85 |
| 9 | 15:10 | 44:03 | 16.8 |
| 10 | 42:01 | 44:03 | 0.35 |
| 11 | 44:03 | 58:01 | 2.39 |
| 12 | 42:01 | 44:03 | 1.51 |
| 13 | 44:03 | 81:01 | 7.81 |
| 14 | 27:05 | 44:03 | 1.3 |
| 15 | 15:01 | 44:03 | 6.02 |
| 16 | 44:03 | 45:01 | 0.7 |
| 17 | 42:01 | 44:03 | 0.09 |
| 18 | 44:03 | 58:01 | 4.49 |
| 19 | 08:01 | 44:03 | 2.18 |
| 20 | 42:01 | 44:03 | 0.08 |
