# Supplementary material for: Major TCR Repertoire Perturbation by Immunodominant HLA-B*44:03-Restricted CMV-Specific T Cells
Source: Front Immunol. 2018 Nov 14;9:2539. doi: 10.3389/fimmu.2018.02539 (PMC6246681; doi:10.3389/fimmu.2018.02539)
Supplement: Supplementary Table 2 — Public TCR clonotypes are encoded by multiple nucleotide sequences. The nucleotide and amino acid sequence of superdominant public clonotypes is shown, together with their absolute read count and relative frequency. [file Table_2.docx]

| **Sample ID** | **Read Count** | | **Read %** | **CDR3 nucleotide** | **SEQ QUALITY** | **V GENE** | **J GENE** | **CDR3 aa** |
| --- | --- | --- | --- | --- | --- | --- | --- | --- |
| 80 | 1580 | | 91.17% | TGTGCCAGTAGTATTTTCGGTGAGCAGTTCTTC | SSSSSSSSSSSSSSSSSSSSSSSSSSSSSSSSS | TRBV19 | TRBJ2-1 | CASSIFGEQFF |
| 83 | 177 | | 8.01% | TGTGCCAGTAGTATTTTCGGTGAGCAGTTCTTC | SSSSSSSSSSSSSSSSSSSSSSSSSSSSSSSSS | TRBV19 | TRBJ2-1 | CASSIFGEQFF |
| 203 | 36 | | 16.51% | TGTGCCAGTAGTATCTTTGGGGAGCAGTTCTTC | SSSSSSSSSSSSSSSSSSSSSSSSSSSSSSSSS | TRBV19 | TRBJ2-1 | CASSIFGEQFF |
| 232 | 36 | | 16.51% | TGTGCCAGTAGTATCTTTGGGGAGCAGTTCTTC | SSSSSSSSSSSSSSSSSSSSSSSSSSSSSSSSS | TRBV19 | TRBJ2-1 | CASSIFGEQFF |
| 81 | 3 | | 0.22% | TGTGCCAGTAGTATTTTTGGTGAGCAGTTCTTC | HHHHHHHHHHHHHHHHHHHHHHHHHHHHHHH | TRBV19 | TRBJ2-1 | CASSIFGEQFF |
|  |  | |  |  |  |  |  |  |
| **Sample ID** | **Read Count** | | **Read %** | **CDR3 nucleotide** | **SEQ QUALITY** | **V GENE** | **J GENE** | **CDR3 aa** |
| 162 | 448 | | 6.22% | TGTGCCAGTAGTATCTTCGGGGAGCTGTTTTTT | SSSSSSSSSSSSSSSSSSSSSSSSSSSSSSSSS | TRBV19 | TRBJ2-2 | CASSIFGELFF |
| 58 | 381 | | 6.50% | TGTGCCAGTAGTATTTTTGGGGAGCTGTTTTTT | SSSSSSSSSSSSSSSSSSSSSSSSSSSSSSSSS | TRBV19 | TRBJ2-2 | CASSIFGELFF |
| 58 | 42 | | 0.72% | TGTGCCAGTAGTATCTTCGGGGAGCTGTTTTTT | SSSSSSSSSSSSSSSSSSSSSSSSSSSSSSSSS | TRBV19 | TRBJ2-2 | CASSIFGELFF |
|  |  | |  |  |  |  |  |  |
| **Sample ID** | **Read Count** | | **Read %** | **CDR3 nucleotide** | **SEQ QUALITY** | **V GENE** | **J GENE** | **CDR3 aa** |
| 30 | 10117 | | 15.31% | TGTGCTGTGGGGAATAATGCAGGCAACATGCTCACCTTT | SSSSSSSSSSSSSSSSSSSSSSSSSSSSSSSSSSSSSSS | TRAV20 | TRAJ39 | CAVGNNAGNMLTF |
| 30 | 1231 | | 1.86% | TGTGCTGTGGGCAATAATGCAGGCAACATGCTCACCTTT | SSSSSSSSSSSSSSSSSSSSSSSSSSSSSSSSSSSSSSS | TRAV20 | TRAJ39 | CAVGNNAGNMLTF |
| 81 | 9 | | 0.04% | TGTGCTGTGGGGAATAATGCAGGCAACATGCTCACCTTT | HHHHHGGHHHHHHHHHHHHHHHHHHHHHHHHHHHHH | TRAV20 | TRAJ39 | CAVGNNAGNMLTF |
| 443 | 8211 | | 41.57% | TGTGCTGTGGGCAATAATGCAGGCAACATGCTCACCTTT | SSSSSSSSSSSSSSSSSSSSSSSSSSSSSSSSSSSSSSS | TRAV20 | TRAJ39 | CAVGNNAGNMLTF |
| 443 | 4660 | | 23.59% | TGTGCTGTGGGTAATAATGCAGGCAACATGCTCACCTTT | SSSSSSSSSSSSSSSSSSSSSSSSSSSSSSSSSSSSSSS | TRAV20 | TRAJ39 | CAVGNNAGNMLTF |
| 204 | 11 | | 0.19% | TGTGCTGTGGGTAATAATGCAGGCAACATGCTCACCTTT | HHHHHGGHHHHHHHHHHHHHHHHHHHHHHHHHHHHH | TRAV20 | TRAJ39 | CAVGNNAGNMLTF |
| 243 | 7 | | 2.73% | TGTGCTGTGGGGAATAATGCAGGCAACATGCTCACCTTT | HHHHHGGHHHHHHHHHHHHHHHHHHHHHHHHHHHHH | TRAV20 | TRAJ39 | CAVGNNAGNMLTF |
| 203 | 3259 | | 23.71% | TGTGCTGTGGGTAATAATGCAGGCAACATGCTCACCTTT | SSSSSSSSSSSSSSSSSSSSSSSSSSSSSSSSSSSSSSS | TRAV20 | TRAJ39 | CAVGNNAGNMLTF |
| 203 | 2249 | | 16.36% | TGTGCTGTGGGCAATAATGCAGGCAACATGCTCACCTTT | SSSSSSSSSSSSSSSSSSSSSSSSSSSSSSSSSSSSSSS | TRAV20 | TRAJ39 | CAVGNNAGNMLTF |
| 52 | 2328 | | 100.00% | TGTGCTGTGGGAAATAATGCAGGCAACATGCTCACCTTT | SSSSSSSSSSSSSSSSSSSSSSSSSSSSSSSSSSSSSSS | TRAV20 | TRAJ39 | CAVGNNAGNMLTF |
| 41 | 249 | | 16.05% | TGTGCTGTCGGGAATAATGCAGGCAACATGCTCACCTTT | SSSSSSSSSSSSSSSSSSSSSSSSSSSSSSSSSSSSSSS | TRAV20 | TRAJ39 | CAVGNNAGNMLTF |
| 41 | 88 | | 5.67% | TGTGCTGTGGGAAATAATGCAGGCAACATGCTCACCTTT | SSSPSLSSSSSSSSSSSSSSSRSSSSSSSSSPSSSSLSS | TRAV20 | TRAJ39 | CAVGNNAGNMLTF |
| 58 | 57421 | | 100.00% | TGTGCTGTGGGGAATAATGCAGGCAACATGCTCACCTTT | SSSSSSSSSSSSSSSSSSSSSSSSSSSSSSSSSSSSSSS | TRAV20 | TRAJ39 | CAVGNNAGNMLTF |
| 83 | 245 | | 6.25% | TGTGCTGTGGGGAATAATGCAGGCAACATGCTCACCTTT | SSSSSSSSSSSSSSSSSSSSSSSSSSSSSSSSSSSSSSS | TRAV20 | TRAJ39 | CAVGNNAGNMLTF |
| 20 | 10840 | 16.05% | | TGTGCTGTCGGGAATAATGCAGGCAACATGCTCACCTTT | SSSSSSSSSSSSSSSSSSSSSSSSSSSSSSSSSSSSSSS | TRAV20 | TRAJ39 | CAVGNNAGNMLTF |
|  |  |  | |  |  |  |  |  |
| **Sample ID** | **Read Count** | **Read %** | | **CDR3 nucleotide** | **SEQ QUALITY** | **V GENE** | **J GENE** | **CDR3 aa** |
| 30 | 3508 | 5.31% | | TGTGCTGTGGGGGCGAATGCAGGCAACATGCTCACCTTT | SSSSSSSSSSSSSSSSSSSSSSSSSSSSSSSSSSSSSSS | TRAV20 | TRAJ39 | CAVGANAGNMLTF |
| 232 | 32 | 0.19% | | TGTGCTGTGGGGGCGAATGCAGGCAACATGCTCACCTTT | HHHHHGGGGGGGGHHHHHHHHHHHHHHHHHHHHHH | TRAV20 | TRAJ39 | CAVGANAGNMLTF |
| 204 | 3146 | 54.17% | | TGTGCTGTGGGGGCGAATGCAGGCAACATGCTCACCTTT | SSSSSSSSSSSSSSSSSSSSSSSSSSSSSSSSSSSSSSS | TRAV20 | TRAJ39 | CAVGANAGNMLTF |
| 243 | 17 | 6.64% | | TGTGCTGTGGGGGCGAATGCAGGCAACATGCTCACCTTT | SSSSSSSSSSSSSSSSSSSSSSSSSSSSSSSSSSSSSSS | TRAV20 | TRAJ39 | CAVGANAGNMLTF |
| 203 | 8 | 0.06% | | TGTGCTGTGGGGGCGAATGCAGGCAACATGCTCACCTTT | HHHHHGGGGGGGGHHHHHHHHHHHHHHHHHHHHHH | TRAV20 | TRAJ39 | CAVGANAGNMLTF |
| 206 | 4660 | 26.32% | | TGTGCTGTGGGGGCGAATGCAGGCAACATGCTCACCTTT | SSSSSSSSSSSSSSSSSSSSSSSSSSSSSSSSSSSSSSS | TRAV20 | TRAJ39 | CAVGANAGNMLTF |
| 64 | 17421 | 100.00% | | TGTGCTGTGGGGGCGAATGCAGGCAACATGCTCACCTTT | SSSSSSSSSSSSSSSSSSSSSSSSSSSSSSSSSSSSSSS | TRAV20 | TRAJ39 | CAVGANAGNMLTF |

**Supplementary Table 2. Public TCR clonotypes are encoded by multiple nucleotide sequences.** The nucleotide and amino acid sequence of superdominant public clonotypes is shown, together with their absolute read count and relative frequency.
