# Supplementary material for: Major TCR Repertoire Perturbation by Immunodominant HLA-B*44:03-Restricted CMV-Specific T Cells
Source: Front Immunol. 2018 Nov 14;9:2539. doi: 10.3389/fimmu.2018.02539 (PMC6246681; doi:10.3389/fimmu.2018.02539)
Supplement: Supplementary Table 3 — TCR-α chain clonotypes are shared amongst HLA-B*44:03+ individuals and encoded by multiple nucleotide sequences. TCR-α chain sequencing from genomic DNA was carried out in three HLA-B*44:03+ individuals, using the Immunoseq platform (Adaptive Biotechnologies). Nucleotide and amino acid sequences are shown, together with their absolute count and relative frequency. [file Table_3.DOCX]

| **Sample ID** | **Read Count** | **Frequency** | **CDR3 nucleotide** | **V GENE** | **J GENE** | **CDR3 aa** |
| --- | --- | --- | --- | --- | --- | --- |
| 09-0193 | 6945 | 3.344 | GTGCTGTCGGGAATAAT | TRAV20 | TRAJ39 | CAVGNNAGNMLTF |
|  |  |  |  |  |  |  |
| 09-0207 | 6972 | 1.52 | GTGCTGTGGGCAATAAT | TRAV20 | TRAJ39 | CAVGNNAGNMLTF |
| 09-0207 | 3654 | 0.796 | GTGCTGTGGGGAATAAT | TRAV20 | TRAJ39 | CAVGNNAGNMLTF |
| 09-0207 | 8 | 0.002 | GTGCTGTTGGTAATAAT | TRAV20 | TRAJ39 | CAVGNNAGNMLTF |
| 09-0207 | 7 | 0.002 | GTGCTGTGGGTAATAAT | TRAV8-3 | TRAJ39 | CAVGNNAGNMLTF |
|  |  |  |  |  |  |  |
| 09-0195 | 68 | 0.02 | GTGCTGTTGGGAATAAT | TRAV20 | TRAJ39 | CAVGNNAGNMLTF |
| 09-0195 | 9 | 0.003 | GTGCTGTCGGTAATAAT | TRAV20 | TRAJ39 | CAVGNNAGNMLTF |
|  |  |  |  |  |  |  |
| 09-0195 | 3800 | 1.082 | GTGCTGTGGGGGCTAAT | TRAV20 | TRAJ39 | CAVGANAGNMLTF |

**Supplementary Table 3. TCR-α chain clonotypes are shared amongst HLA-B*44:03+ individuals and encoded by multiple nucleotide sequences.** TCR-α chain sequencing from genomic DNA was carried out in three HLA-B*44:03+ individuals, using the Immunoseq platform (Adaptive Biotechnologies). Nucleotide and amino acid sequences are shown, together with their absolute count and relative frequency.
